# Antibacterial Effect and Possible Mechanism of Salicylic Acid Microcapsules against *Escherichia coli* and *Staphylococcus aureus*

**DOI:** 10.3390/ijerph191912761

**Published:** 2022-10-06

**Authors:** Xiaoqiu Song, Rui Li, Qian Zhang, Shoukui He, Yifei Wang

**Affiliations:** 1 Department of Food Science and Technology, School of Perfume and Aroma Technology, Shanghai Institute of Technology, Shanghai 201418, China; 2 Department of Food Science and Technology, School of Agriculture and Biology, Shanghai Jiao Tong University, Shanghai 200240, China

**Keywords:** salicylic acid, β-cyclodextrin, microcapsule, antibacterial activity, antibacterial mechanism, *Escherichia coli*, *Staphylococcus aureus*

## Abstract

Microcapsules serve as a feasible formulation to load phenolic substances such as salicylic acid, a natural and safe antimicrobial agent. However, the antibacterial efficacy of salicylic acid microcapsules (SAMs) remains to be elucidated. Here, salicylic acid/β-cyclodextrin inclusion microcapsules were subjected to systematic antibacterial assays and preliminary antibacterial mechanism tests using *Escherichia coli* and *Staphylococcus aureus* as target organisms. It was found that the core-shell rhomboid-shaped SAMs had a smooth surface. SAMs exhibited a minimum inhibitory concentration (MIC) and a minimum bactericidal concentration (MBC) of 4 mg/mL against both bacteria. In the growth inhibition assay, 1/4 × MIC, 1/2 × MIC, and 1 × MIC of SAMs effectively retarded bacterial growth, and this effect was more prominent with the rise in the level of SAMs. Practically, SAMs possessed a rapid bactericidal effect at the 1 × MIC level with a reduction of more than 99.9% bacterial population within 10 min. A pronounced sterilization activity against *E. coli* and *S. aureus* was also observed when SAMs were embedded into hand sanitizers as antimicrobial agents. Moreover, exposure of both bacteria to SAMs resulted in the leakage of intracellular alkaline phosphatases and macromolecular substances (nucleic acids and proteins), which indicated the disruption of bacterial cell walls and cell membranes. In conclusion, SAMs were able to inactivate *E. coli* and *S. aureus* both *in vitro* and *in situ*, highlighting the promising utilization of this formulation for antimicrobial purposes in the area of food safety and public health.

## 1. Introduction

The growing demand for food safety has motivated the search for natural and safe antimicrobial substances to control pathogenic bacteria such as *Staphylococcus aureus* and *Escherichia coli* in the food industry. Salicylic acid derived from natural willow bark and other plants is a kind of safe agent which exerts a high potential for antimicrobial purposes during food processing and preservation [1]. A large number of scholars have focused on the antifungal efficiency of salicylic acid, since this natural compound can be produced by plants in response to microbial invasions [2,3]. Indeed, salicylic acid is effective in controlling postharvest fungal pathogens such as *Penicillium expansum*, *Botrytis cinerea*, *Fusarium oxysporum*, and *Rhizopus stolonifer* in fruit and vegetables [4,5,6]. Moreover, several studies have illustrated the inhibitory activity of starch- or chitosan-based films incorporated with salicylic acid as an active agent against bacterial strains such as *S. aureus*, *E. coli*, and *Bacillus subtilis* by a qualitative disc diffusion method [1,7]. To date, however, no quantitative studies (e.g., minimum inhibitory concentration tests and time-kill assays) have been conducted on the antibacterial activity of salicylic acid-based formulations. Moreover, the main disadvantage centers around low water solubility when salicylic acid is applied to foods. This makes further utilization of salicylic acid a bit challenging in the food industry.

Encapsulation serves as a strategy to counteract the low water solubility of phenolic substances, such as salicylic acid. This technology can entrap solid particles, liquid droplets, or gaseous compounds in wall materials, thus providing an effective approach for controlled release and protection of active compounds [8]. In the food and pharmaceutical industries, chitosan, sodium alginate, and β-cyclodextrin (β-CD) are commonly used as wall materials for encapsulating hydrophobic core substances [9,10,11]. Among these wall materials, β-CD is considered as an ideal one owing to its low cost, good thermal stability, and commercial development prospects [12]. Recently, molecular inclusion in β-CD complexes has been reported to be effective in masking the undesirable smell of essential oils and increasing their solubility [13].

Preliminary work in our group has demonstrated the successful preparation of salicylic acid/β-CD inclusion complexes and the resulting salicylic acid microcapsules (SAMs) exhibited appreciable water solubility. To our knowledge, there is currently no relevant study on the antibacterial activity of β-CD inclusion complexes containing salicylic acid, a safe and natural phenolic compound. Practically, it has been revealed that other phenolic substances can accumulate at the bacterial surface and disrupt cell membranes and cell walls [14]. Therefore, analysis of bacterial surface properties will contribute to a better understanding of the mode of action of SAMs. In this context, the aim of the current work was to elucidate the inhibitory activity of SAMs against *S. aureus* and *E. coli* via systematic antibacterial tests coupled with cell property analyses, which may help to broaden the application of salicylic acid in the food industry.

## 2. Materials and Methods

### 2.1. Materials

Nutrient agar (NA) and nutrient broth (NB) were purchased from Qingdao Hope Bio-Technology Co., Ltd., Qingdao, Shandong, China. Analytical grade ethanol was supplied by Shanghai Richjoint Chemical Reagents Co. Ltd., Shanghai, China. Salicylic acid, β-CD, sodium chloride, and other chemicals were obtained from Sinopharm Chemical Reagent Co., Ltd., Shanghai, China.

### 2.2. Test Strains

The target bacteria were Gram-negative *E. coli* ATCC 25922 and Gram-positive *S. aureus* ATCC 25923. Before testing, each strain was inoculated in NA at 37 °C for 24 h and then transferred to NB, followed by incubation at 37 °C for 24 h. Subsequently, bacterial suspensions were centrifuged (TGL-16M, Cence, Changsha, Hunan, China) at 2000 rpm for 3 min. The pellets were diluted in sterile saline to prepare bacterial suspensions with a final concentration of 5 × 10^8^ CFU/mL, which were used in subsequent experiments.

### 2.3. Preparation of SAMs

According to the Patent CN108210346B, salicylic acid/β-CD inclusion microcapsules were prepared [15]. This host-guest inclusion complex can be formed since the sugar structures of β-CD with hydrophobic cavities and hydrophilic exteriors allow fat-soluble salicylic acid to be encapsulated. Salicylic acid is stable in the inclusion complex due to the fact that its carboxyl group is located in the plane of the upper edge of β-CD molecule, and a weak hydrogen bond is formed between phenyl hydroxyl and glycoside oxygen [16]. A total of 7.5 g β-CD was dissolved in 100 mL distilled water (95 °C) to form a wall material solution. Salicylic acid (2.3 g) was dissolved in 0.4 mL ethanol at 20 °C and then slowly added to the wall material solution. The resulting mixture was continuously stirred at 500 rpm and 50 °C for 24 h to obtain the microcapsule solution. The solution was then cooled to 20 °C, filtrated, washed with deionized water at least five times, and dried at 50 °C for 2 h to prepare white SAM powders.

The effect of storage temperature on microcapsule stability was then assessed. The formulated SAMs were added to 20 mL NB in centrifuge tubes to reach final concentrations of 2 to 8 mg/mL. Tubes were then stored at different temperatures (−4 °C, 37 °C, and 40 °C) for 24 h. It was observed that all concentrations of SAMs retained transparent without precipitation or layer separation in NB.

### 2.4. Characterization of Morphology

The morphological characteristics of SAMs were analyzed using a transmission electron microscope (TEM) (Tecnai G2 F20 S-TWIN, FEI, Hillsboro, USA). Briefly, SAM dispersions (1 mg/mL in distilled water) were prepared, dropped onto 200 meshes of carbon-coated copper grid, and then air-dried. TEM measurements were carried out at 200 kV at room temperature (25 °C).

### 2.5. Assessment of Loading Capacity

The loading capacity of SAMs was determined using UV-Vis spectroscopy (UV1800, Shimadzu, Kyoto, Japan) at a wavelength of 300 nm according to a modified version of methods proposed by Piletti et al. [11]. A standard calibration curve was obtained based on the absorbance of salicylic acid dilutions (10–30 μg/mL) in ethanol. Subsequently, 0.2 g of SAMs was added to 100 mL of absolute ethanol. After 24 h, the mixture was centrifuged at 4000 rpm for 5 min. The absorbance of the supernatant containing salicylic acid was measured, and the loading capacity of SAMs was calculated as follows:

Loading capacity = (the mount of encapsulated salicylic acid)/(the amount of microcapsules) × 100%(1)

### 2.6. Determination of Minimum Inhibitory and Bactericidal Concentrations

Minimum inhibitory concentration (MIC) is an indicator of the antimicrobial strength of active components, while minimum bactericidal concentration (MBC) represents the minimum concentration required to kill 99.9% of the tested microorganisms [17]. Thus, lower MIC and MBC values correspond to stronger antibacterial and bactericidal abilities. The MIC and MBC values of SAMs against *E. coli* and *S. aureus* were determined through the two-fold serial dilution method [18]. Briefly, the prepared bacterial suspensions were added to NB, followed by the addition of SAMs to a final concentration of 2 to 16 mg/mL. Then, bacterial suspensions were incubated at 37 °C with shaking at 200 rpm for 24 h. The minimum concentration at which the visible growth of the target strain was completely inhibited was considered the MIC value. In contrast, the minimum concentration to completely kill the microorganisms was considered the MBC value. The experiments were repeated twice, with three replicates for each concentration.

### 2.7. Growth Inhibition Tests

The effect of SAMs on bacterial proliferation in *E. coli* and *S. aureus* was determined using UV-Vis spectroscopy at a wavelength of 600 nm [19]. Bacterial suspensions were added to NB supplemented with 0 ×, 1/4 ×, 1/2 ×, and 1 × MIC of SAMs. The growth of bacteria was determined after incubation at 37 °C with shaking at 200 rpm. The absorbance was determined every 2 h and the antibacterial dynamic curves were obtained from the data. All experiments were performed in triplicate.

### 2.8. Time-Kill Assays

The bactericidal effect of SAMs was evaluated using a time-kill assay [20]. Briefly, SAMs were added to different bacterial suspensions to the final level of 1 × MIC, which were then shaken at 200 rpm (37 °C). Bacterial colonies were determined after the bacterial suspensions were exposed to SAMs for 0, 0.25, 1, and 10 min by the plate count method. Sterile distilled water was used as a control group in a similar way. All experiments were performed in triplicate.

### 2.9. Antibacterial Efficacy of SAMs in Hand Sanitizers

Control hand sanitizers were obtained from the International School of Cosmetics at Shanghai Institute of Technology, China, with inactive ingredients including cocamidopropyl betaine, alkyl polyglucoside, cocamide DEA, 1,3-butylene glycol, glycerin, disodium EDTA, PEG-14M, and sodium chloride. SAM-based hand sanitizers were prepared with the addition of SAMs to the dose of 1/2 ×, 1 ×, and 2 × MIC, respectively. Briefly, either *E. coli* or *S. aureus* was incubated in NB at 37 °C overnight, and then adjusted to a bacterial concentration of 1 × 10^7^ CFU/mL. Approximately 1 g hand sanitizers were transferred to a conical flask containing 9 mL bacterial suspensions. Samples were vortexed for 0, 0.25, 1, and 10 min, respectively, and 2% lecithin was added as a neutralizer. Serial dilutions were spread on the surface of NA and then cultivated at 37 °C for 18–24 h. The viable counts were determined in triplicate for each decimal dilution.

### 2.10. Analysis of Cell Wall Permeability

The integrity of cell walls can be judged by alkaline phosphatase (AKP) activity, which was detected according to the method of Wang et al. [21] with slight modifications. The bacterial suspension was washed with PBS three times and then resuspended. Then, SAMs were added to achieve a final concentration of 1/2 × MIC and 1 × MIC. The samples were cultured at 37 °C for 0, 0.25, 1, and 10 min, and then 0.2 mL filtrates with 0.22 μm filter membrane were added to 1.8 mL of 0.1 mol/L Tris-HCl buffer (pH 8.0, containing 0.2 mg/mL 4-nitrophenyl phosphate disodium salt). Afterwards, mixtures were incubated at 25 °C for 30 min. The absorbance value at 410 nm was used to express the content of AKP in the solution. The blank control group was treated with sterile distilled water.

### 2.11. Measurement of Cell Membrane Permeability

Membrane permeability was determined by measuring the leakage of nucleic acids and proteins from the bacteria [22,23]. The bacterial suspension was washed with PBS three times, and exposed to SAMs dilutions with the final concentrations of 1/2 × MIC and 1 × MIC. After incubation at 37 °C and 120 rpm for 0, 0.25, 1, and 10 min, 5 mL of bacterial suspensions were removed and passed through a 0.22 μm filter. The content of nucleic acids in the bacterial suspensions was determined by the absorbance value of the filtrate at 260 nm. Similarly, the content of soluble proteins after filtration was determined by the Coomassie Blue Staining. The filtrates (1 mL) of all samples were thoroughly mixed with Coomassie brilliant blue (G-250) dye solutions (5 mL) for 2 min, and the absorbance of all solutions at 595 nm was measured. The blank control group was treated with sterile distilled water.

### 2.12. Data Analysis

Analysis of variance was performed using SAS 9.4 software. Duncan’s multiple comparison method was used to detect statistically significant differences at the *p* < 0.05 level.

## 3. Results and Discussion

### 3.1. TEM Morphology of SAMs

**Figure 1:**
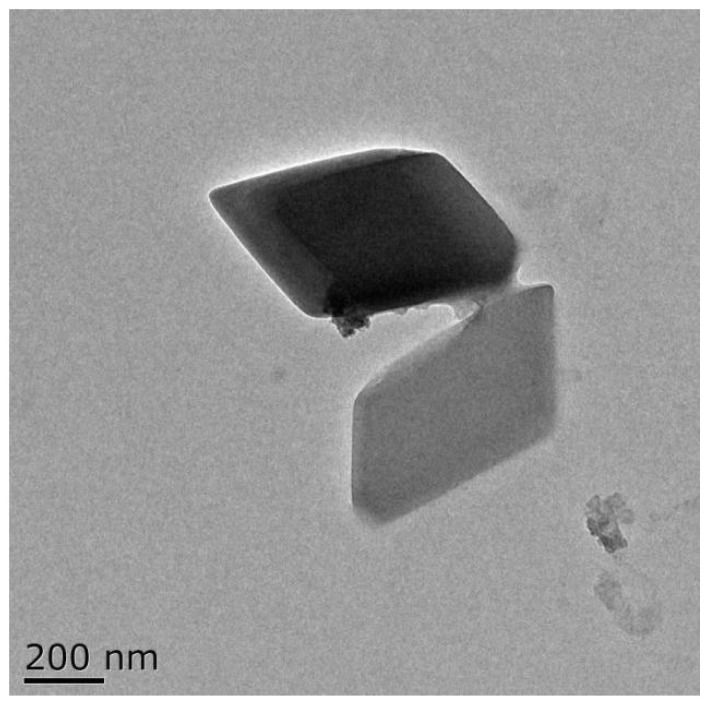
TEM images of SAMs. Scale bars correspond to 200 nm.

As shown in Figure 1, salicylic acid/β-CD inclusion microcapsules had an obvious core-shell rhomboid shape and a smooth surface with a diameter ranging from 0.5 to 0.6 μm. The clear β-CD shells around the salicylic acid sphere, with the wall thickness in the range of 40–60 nm, strongly confirmed the successful encapsulation of salicylic acid in β-CD. A similar result was reported by Anaya–Castro et al. [24], who found that clove essential oil could be embedded by β-CD to form a core-shell rhomboid structure.

The formation and shape of microcapsules are affected by many factors such as preparation methods, type of core and wall materials, and stirring speeds [25,26]. Similarly, other delivery systems such as emulsions with desirable characteristics can be produced by adjusting emulsifier type, continuous or dispersed phase concentration, or the emulsification process, etc [27,28]. Hence, control of fabrication process parameters is of great importance for the design of suitable delivery systems for food applications.

### 3.2. Loading Capacity of SAMs

Loading capacity has been widely used to determine the amount of encapsulated core materials [29]. In this work, the loading capacity of SAMs in β-CD was determined using a calibration curve based on the UV absorbance values. The loading capacity of SAMs was found to be 26.7% after the encapsulation processes. This showed that a low-molecular-weight organic acid such as salicylic acid was successfully encapsulated in β-CD using the host-guest inclusion complexation method. Similarly, cinnamon essential oil was embedded into a microcapsule with a high loading capacity of 30.2% in our previous work [30].

### 3.3. MIC and MBC Values of SAMs

The MIC and MBC tests were employed as a quantitative method to assess the antibacterial activity of SAMs. It was observed that SAMs exhibited both MIC and MBC values of 4 mg/mL against *S. aureus* and *E. coli*. In previous work, cinnamon essential oil displayed pronounced antibacterial performance against a variety of bacterial strains (e.g., *S. aureus* and *E. coli*), with MIC values ranging from 2.5 to 5 mg/mL, and MBC values ranging from 2.5 to 10 mg/mL [31]. It was thus indicated that SAMs in this work possessed excellent antibacterial properties comparable to cinnamon essential oil, whose potential for antibacterial applications in the food industry has been well documented. In this sense, future application of SAMs during food processing and preservation to ensure food safety can be anticipated.

It should be noted that the *in vitro* antibacterial activity of salicylic acid-based formulations has been only previously assessed qualitatively by a disc diffusion method [1,7]. For instance, chitosan films enriched with salicylic acid showed an inhibition zone diameter between 6 and 8 mm against *S. aureus* and *E. coli* [7]. Interestingly, our current work employed a quantitative approach to determine the bacteriostatic and bactericidal effects of SAMs, which enforces our knowledge on the antibacterial performance of salicylic acid on bacterial strains.

### 3.4. Growth Inhibition Activity of SAMs

**Figure 2:**
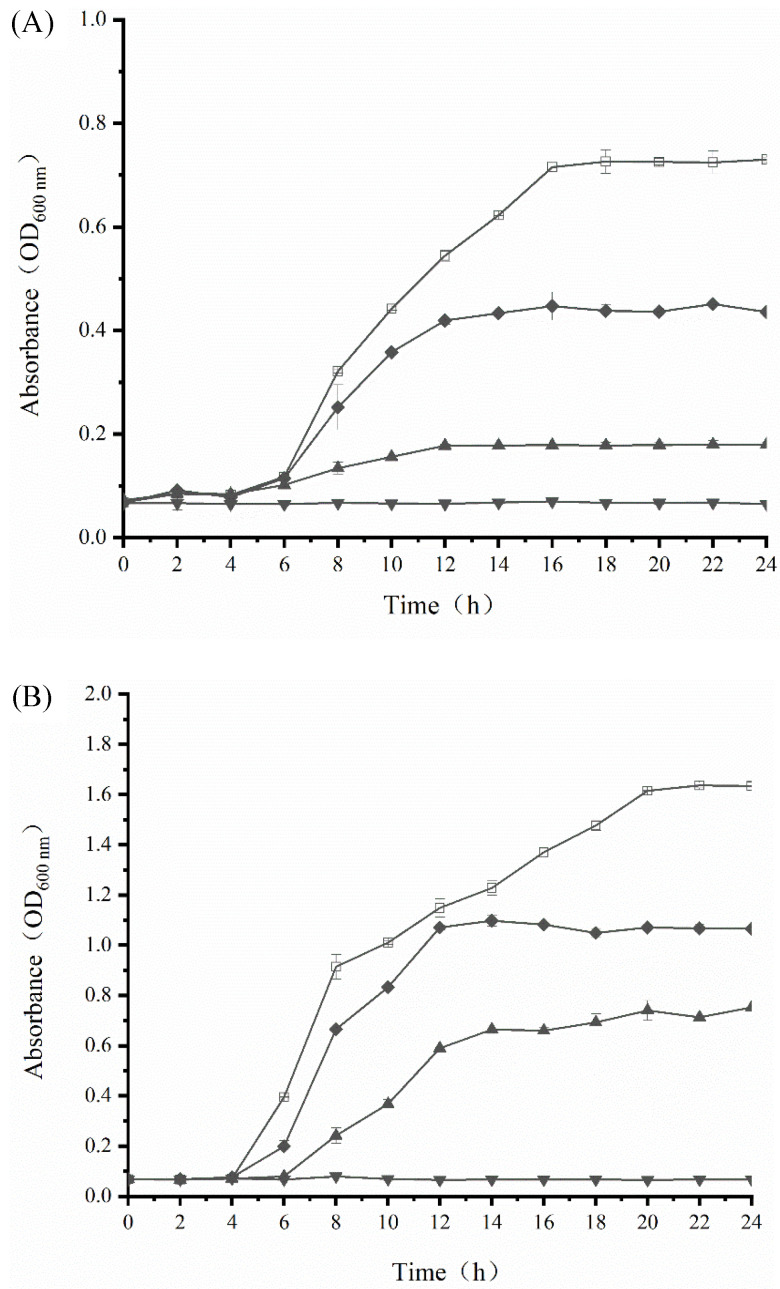
Growth curve of *E. coli* (**A**) and *S. aureus* (**B**) during exposure to different concentrations of SAMs in NB at 37 °C for 24 h. The following concentrations of SAMs were used: control (□), 1/4 × MIC (◆), 1/2 × MIC (▲), and 1 × MIC (▼). NB, nutrient broth. Values are displayed as the mean ± standard deviation. Error bars show standard deviations, which have been plotted for all data points. Symbols without visible vertical bars suggest that the symbol size is larger than the standard deviation.

In order to further explore the bacteriostatic activity of SAMs, dynamic growth curves of *S. aureus* and *E. coli* were determined under different concentrations of SAMs (0 ×, 1/4 ×, 1/2 ×, and 1 × MIC) (Figure 2). Both bacterial strains in the control group showed a rapid increase in OD_600_ values, reaching a steady state after 16 h for *E. coli*, and 20 h for *S. aureus*, respectively. Treatments with SAMs resulted in reduced bacterial growth, and this inhibitory effect was more pronounced with the rise in the concentration of SAMs. When SAMs were applied at the 1 × MIC level, a complete inhibition in bacterial growth was achieved, which confirmed the accuracy of the results derived from the MIC test. Notably, the bacteriostatic activity of SAMs was more prominent in *S. aureus* compared to that in *E. coli*. For example, the OD_600_ value of bacterial suspensions of *S. aureus* and *E. coli* was reduced by approximately 0.9 and 0.5, respectively, after exposure to 1/2 × MIC of SAMs for 24 h.

In agreement with the above mentioned observations, the growth of *S. aureus* and *E. coli* was retarded by 1/2 × MIC of ginger essential oil, and the decline in the OD_600_ value was more pronounced in *S. aureus* (Gram-positive) than *E. coli* (Gram-negative) [32]. The superior growth of *E. coli* under subinhibitory levels of SAMs and essential oils might be related to the thick outer lipopolysaccharide layer of Gram-negative bacteria, which provided protection against antibacterial agents [33,34]. Taken together, SAMs exerted good bacteriostatic activity in a dose-dependent manner, although their effect on Gram-positive and Gram-negative bacteria was different to some extent.

### 3.5. Time-Kill Activity of SAMs

**Figure 3:**
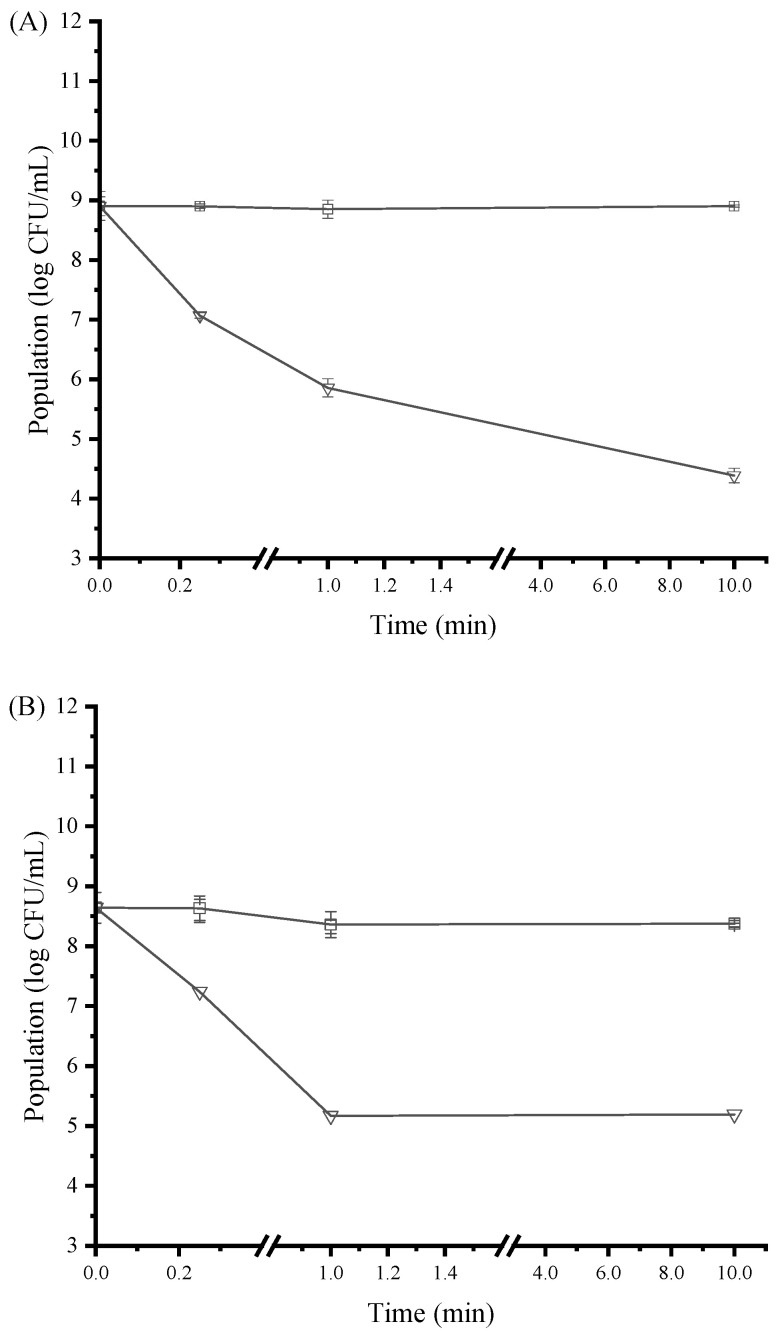
The population of *E. coli* (**A**) and *S. aureus* (**B**) during exposure to different concentrations of SAMs in NB at 37 °C for 10 min. The following concentrations of SAMs were used: control (□) and 1 × MIC (▽). NB, nutrient broth. Values are displayed as the mean ± standard deviation. Error bars show standard deviations, which have been plotted for all data points. Symbols without visible vertical bars suggest that the symbol size is larger than the standard deviation.

A time-kill assay was performed using SAMs at the concentration of 1 × MIC to further assess its bactericidal activity. The bactericidal effects of SAMs were dependent on the duration of contact. As shown in Figure 3A, *E. coli* was gradually killed during exposure to SAMs, reaching a population reduction by approximately 4.5 log CFU/mL after 10 min. In the case of *S. aureus*, the bacterial population was eliminated by approximately 3.5 log CFU/mL within 1 min of contact with SAMs (Figure 3B). These results indicated that SAMs possessed rapid bactericidal effects against both *S. aureus* and *E. coli*.

In previous work, exposure to 4/5 × MIC of cinnamon essential oil for 2 h resulted in a reduction of approximately 99.9% population (i.e., 3-log population reduction) of *S. aureus* and *E. coli* [16]. Given the well-known antibacterial performance of cinnamon essential oil, it can be inferred that the rapid sterilization activity exerted by SAMs in this work was quite satisfactory. The bactericidal action of SAMs could be due to the effective interaction of salicylic acid in the microcapsules with bacteria in the media. Hence, SAMs possessed excellent bacteriostatic and bactericidal capacity, which highlighted the necessity of checking the application potential of SAMs in food, cosmeceutical, and pharmaceutical systems.

### 3.6. Antibacterial Efficacy of SAMs in Hand Sanitizers

As an effective way to prevent the spread of infectious disease, hand washing is attached with increasing importance [35]. Ethanol has been used as an active ingredient in hand sanitizers in food processing plants for a long time [36]. However, bacterial resistance to ethanol and other disinfectants has emerged as a potential food safety concern [37,38]. In this work, SAMs were exploited as an alternative to traditional hand sanitizers.

**Figure 4:**
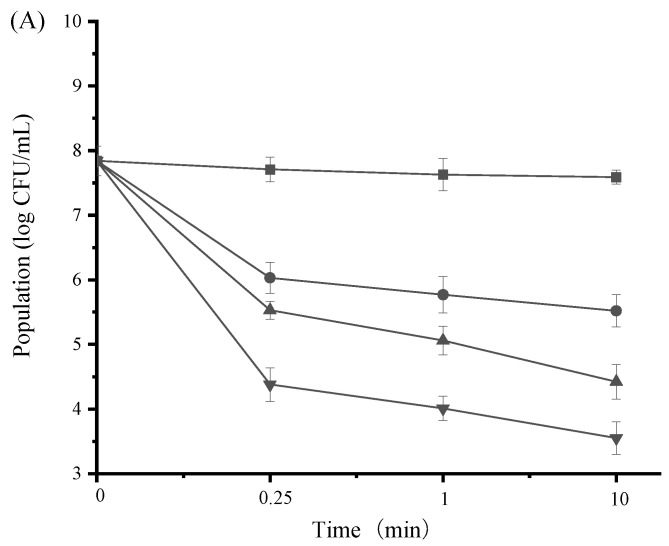

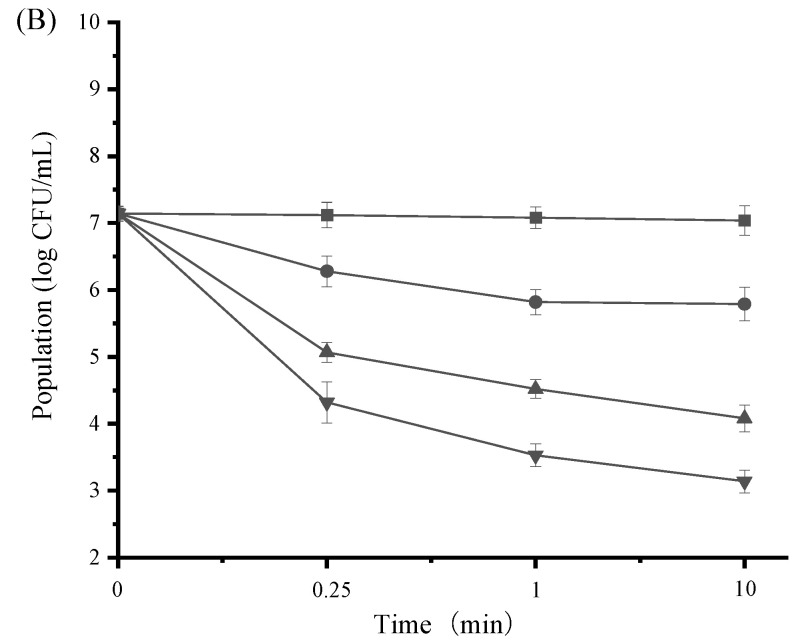
Inhibitory effect of hand sanitizers containing SAMs against *E. coli* (**A**) and *S. aureus* (**B**) during incubation at 37 °C for 10 min. The following concentrations of SAMs were used: control (■), 1/2 × MIC (●), 1 × MIC (▲), and 2 × MIC (▼). Values are displayed as the mean ± standard deviation.

According to Figure 4, the populations of *E. coli* and *S. aureus* were rapidly and progressively reduced at all three tested concentrations. Also, SAMs-based hand sanitizers worked on bacterial strains in a dose-dependent manner. It was observed that the 1 × MIC level of SAMs was able to decrease at least 99% of the initial population (i.e., 2-log population reduction) of *E. coli* and *S. aureus* after 1 min, suggesting that SAMs were active for achieving bactericidal activity in hand sanitizers. Interestingly, a higher concentration (2 × MIC) of SAMs resulted in a reduction of 99.99% of the bacterial populations (i.e., 4-log population reduction) after 10 min. This result probably expands SAMs’ application as washing solutions in sanitizing products and environments.

### 3.7. Impact of SAMs on Bacterial Cell Wall Integrity

**Figure 5:**
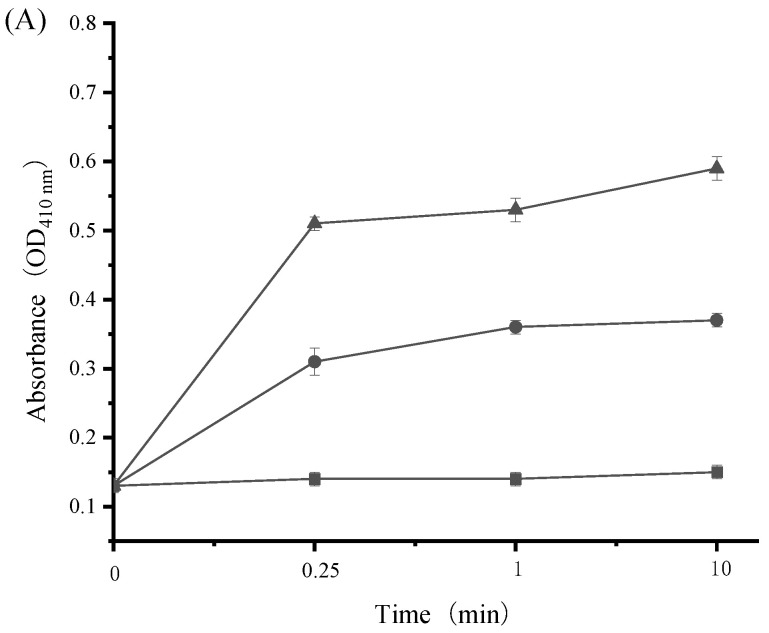

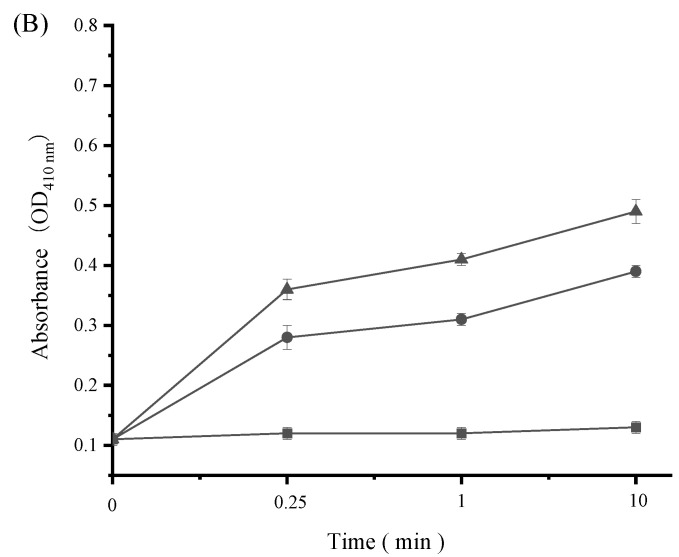
Effect of SAMs on the amount of AKP leakage in *E. coli* (**A**) and *S. aureus* (**B**) during incubation at 37 °C for 10 min. The following concentrations of SAMs were used: control (■), 1/2 × MIC (●), and 1 × MIC (▲). Values are displayed as the mean ± standard deviation. Error bars show standard deviations, which have been plotted for all data points. Symbols without visible vertical bars suggest that the symbol size is larger than the standard deviation.

Bacterial AKP is normally located between cell walls and cell membranes. The activity of this enzyme will not be detected in extracellular environments unless the bacterial cell wall is disrupted [39]. Hence, cell wall damage can be interpreted by the leakage of intracellular AKP into bacterial cell suspensions. As presented in Figure 5, the OD_410_ value of extracellular AKP from both *S. aureus* (approximately 0.11) and *E. coli* (approximately 0.13) was maintained constant in the absence of SAMs. However, this value increased dramatically after *S. aureus* and *E. coli* were treated with SAMs. Furthermore, AKP activity from these two bacterial strains was enhanced along with an increase in SAMs concentration from 1/2 × MIC to 1 × MIC. For example, the OD_410_ value of extracellular AKP in *S. aureus* cell suspensions was increased from 0.11 to 0.39, and 0.11 to 0.49, respectively, in response to 1/2 × MIC and 1 × MIC of SAMs. It can thus be inferred that SAMs may act on the cell wall by disrupting its integrity, which results in the leakage of intracellular AKP into cell suspensions.

In agreement with the above mentioned findings, disruption of cell wall permeability contributed to enhanced extracellular AKP activity after exposure of bacteria to various antibacterial agents [39,40,41,42]. Zhao et al. [42] found that the activity of released AKP increased significantly after *S. aureus* was treated with 2 × MIC of microencapsulated dodecyl gallate with methyl-β-cyclodextrin for 6 h. Similarly, octopus scraps peptides-zinc chelates at the levels of 1/2 × MIC to 1 × MIC could damage the cell wall of *E. coli*, which resulted in the leakage of intracellular AKP [41]. These results indicated that disruption of cell wall integrity might be a key factor leading to bacterial death in the presence of antibacterial agents such as SAMs.

### 3.8. Impact of SAMs on Bacterial Cell Membrane Permeability

**Figure 6:**
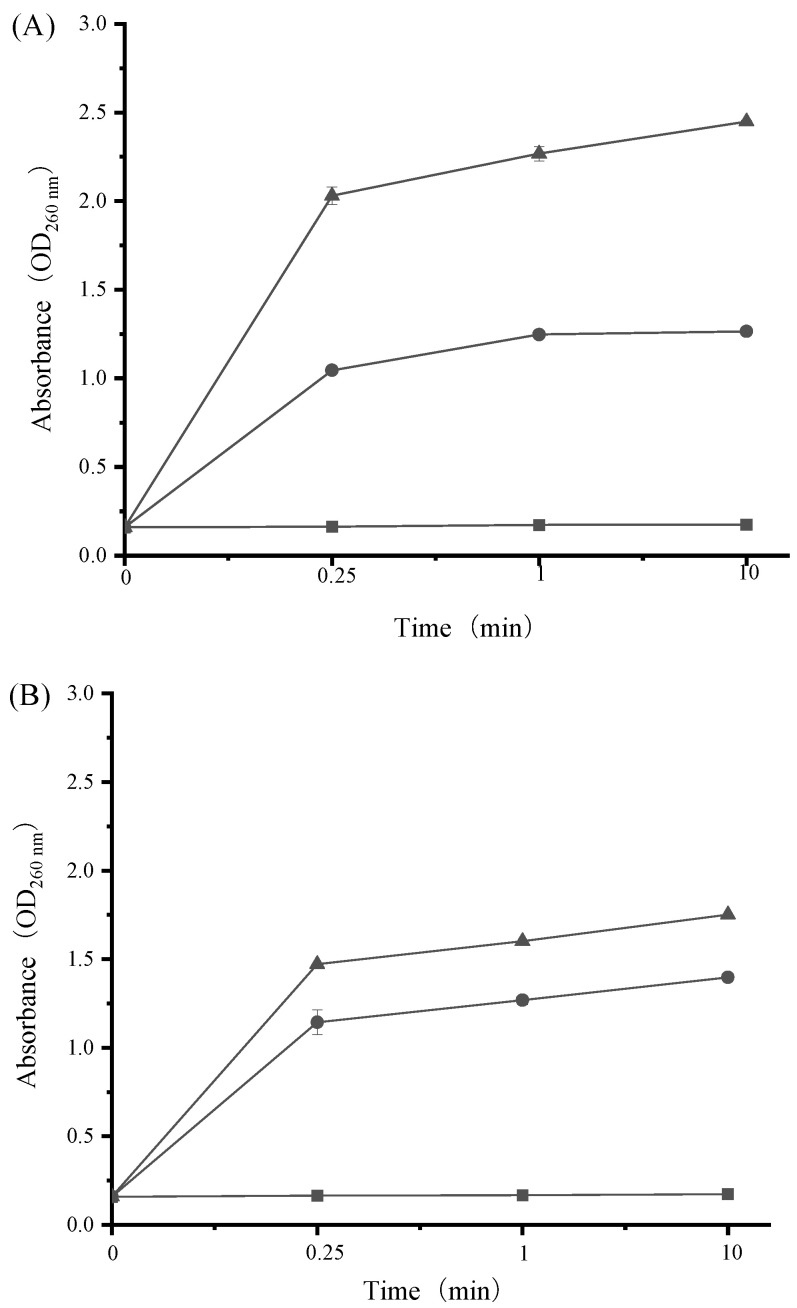
Effect of SAMs on the leakage of intracellular nucleic acids in *E. coli* (**A**) and *S. aureus* (**B**) during incubation at 37 °C for 10 min. The following concentrations of SAMs were used: control (■), 1/2 × MIC (●), and 1 × MIC (▲). Values are displayed as the mean ± standard deviation. Error bars show standard deviations, which have been plotted for all data points. Symbols without visible vertical bars suggest that the symbol size is larger than the standard deviation.

**Figure 7:**
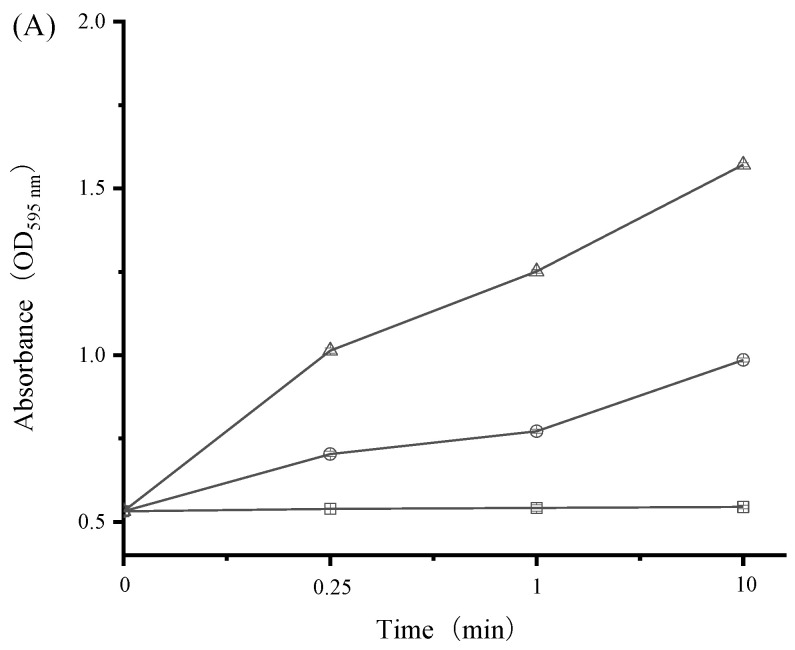

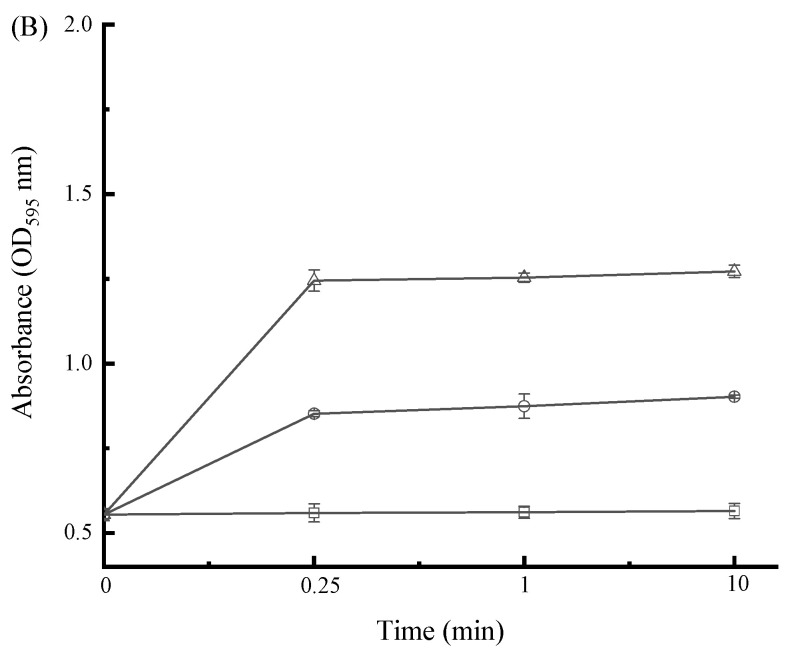
Effect of SAMs on the leakage of intracellular proteins in *E. coli* (**A**) and *S. aureus* (**B**) during incubation at 37 °C for 10 min. The following concentrations of SAMs were used: control (□), 1/2 × MIC (○), and 1 × MIC (△). Values are displayed as the mean ± standard deviation. Error bars show standard deviations, which have been plotted for all data points. Symbols without visible vertical bars suggest that the symbol size is larger than the standard deviation.

Maintenance of cell membrane structure is a prerequisite for bacterial growth and metabolic activity. When the bacterial cell membrane is disrupted, intracellular macromolecular substances (e.g., nucleic acids and proteins) tend to flow out [43,44]. Therefore, the release of cell constituents is a good indicator for assessing bacterial cell membrane integrity in response to antibacterial agents. In the current work, nucleic acids and proteins released from the cytoplasm were monitored by the detection of absorbance at 260 nm and 595 nm, respectively. As shown in Figure 6 and Figure 7, both cell constituents were released rapidly from *S. aureus* and *E. coli* into cell suspensions and their amounts increased multi-fold after treatment with increasing levels of SAMs. For instance, the OD_260_ value of *E. coli* increased significantly from 0.16 for the control to 1.27 and 2.45 in the presence of SAMs at the levels of 1/2 × MIC and 1 × MIC after 10 min, respectively (Figure 6). Moreover, there was a progressive release of proteins from *S. aureus* after exposure to SAMs for 0.25 min, followed by a steady state (Figure 7). These results clearly suggested that bacterial cell membrane integrity was compromised by SAMs, which could consequently result in cell death.

It has been reported that other phenolic substances can also cause disruption in the bacterial cell membrane. Cao et al. [45] found that epigallocatechin gallate could enhance the permeability of the inner membrane and outer membrane in *E. coli* and cause cell membrane depolarization. Similarly, *Kombucha* polyphenolic fraction disrupted the membrane integrity of *Vibrio cholera* as revealed by permeabilization assays, which might be related to the generation of intracellular reactive oxygen species [46]. Therefore, the action of salicylic acid and other phenolic substances on bacterial cell membranes may be due to their interactions with cell membrane proteins, which lead to the loss of chemiosmotic control and finally cell death [47].

## 4. Conclusions

The core-shell rhomboid-shaped SAMs possessed excellent antibacterial performance against both *E. coli* and *S. aureus*, possibly by acting on bacterial cell walls and cell membranes. Notably, a superior sterilization activity was achieved in 10 min with a reduction of more than 99.9% bacterial population. SAMs were also effective in inactivating *E. coli* and *S. aureus* when incorporated into hand sanitizers as active agents. These findings suggest that SAMs have the potential to be utilized as a feasible antibacterial formulation in food, cosmeceutical, and pharmaceutical industries to protect public health. In terms of food applications, the influence of environmental factors (e.g., heat, cold, acid, and salt) on the antibacterial activity of SAMs will be elucidated in future work to further explore the strengths of using this material during food manufacturing. Moreover, it would also be interesting to address the accumulative release of SAMs in the presence of the aforementioned environmental factors related to food processing and preservation.

## Data Availability

All data are available in the article.
